# Image segmentation based on gray level and local relative entropy two dimensional histogram

**DOI:** 10.1371/journal.pone.0229651

**Published:** 2020-03-03

**Authors:** Wei Yang, Lulu Cai, Fei Wu

**Affiliations:** 1 State GRID Quzhou Power Supply Company, No.6, Xinhe Road, Quzhou, Zhejiang 324000, China; 2 College of Electrical and Information Engineering, Quzhou University, Quzhou, Zhejiang 324000, China; Beijing University of Posts and Telecommunications, CHINA

## Abstract

Though traditional thresholding methods are simple and efficient, they may result in poor segmentation results because only image’s brightness information is taken into account in the procedure of threshold selection. Considering the contextual information between pixels can improve segmentation accuracy. To to this, a new thresholding method is proposed in this paper. The proposed method constructs a new two dimensional histogram using brightness of a pixel and local relative entropy of it’s neighbor pixels. The local relative entropy (LRE) measures the brightness difference between a pixel and it’s neighbor pixels. The two dimensional histogram, consisting of gray level and LRE, can reflect the contextual information between pixels to a certain extent. The optimal thresholding vector is obtained via minimizing cross entropy criteria. Experimental results show that the proposed method can achieve more accurate segmentation results than other thresholding methods.

## Introduction

Image segmentation is a fundamental task in many computer vision based applications, such as medical image analysis [1], crack detection [2, 3], video analysis [4], plant disease recognition [5], etc. The main purpose of image segmentation is to categorize an image’s pixels to different classes according to color, texture and brightness, etc. Image segmentation is an active research topic and many segmentation methods had been proposed up to now. The clustering based methods [6–8], regression based methods [9, 10], and deep learning based methods [11–13] are the new and sophisticated methods.

Although the above methods can obtain well segmentation performance, however, the computation complexity and computation burden are relatively high. In practice, simple and effective segmentation methods are desirable. Among different image segmentation methods, thresholding segmentation methods are simple, effective and more easy to be implemented. They become popular and have received much attention of researchers. Thresholding methods assume that there is a deep valley between two peaks in the gray level histogram of the image. The ideal thresholds locate at valleys and can be obtained by optimizing a certain criteria function. The Otsu thresholding method selects the ideal threshold by maximizing the between-class variance between background and objects [14], and Kapur’s thresholding method maximizing the total Shannon entropy of background and objects [15], the Kittler’s thresholding method minimizing the classification error [16]. These classical thresholding methods have some improved variants [17]. However, these classical thresholding segmentation methods and their variants take only the brightness information into account and neglect the contextual information between pixels, which may result in poor segmentation performance or even false segmentation. To solve this problem, Abutaleb proposed the concept of two-dimension histogram [18]. The two-dimension histogram can reflect the contextual information between pixels to a certain extent. By virtue of two-dimension histogram, many classical thresholding methods had been extended to two dimensional case, such as two-dimension Otsu thresholding method [19], two-dimension Tsallis entropy thresholding method [20]. Compared with one-dimension histogram based thresholding methods, two-dimension histogram based thresholding methods can get better segmentation result, especially when the image was corrupted by noise. Unfortunately, it was pointed by Xiao that the two-dimension histogram ignores the edge information of image [21]. In image, edge information is a class of important information which can more effectively reflect contextual information between pixels. Observing this, Xiao proposed a new method to construct two dimension histogram by using the resemblance between a pixel and it’s neighbors as the contextual information and the resulted two dimension histogram is called gray level spatial correlation (GLSC) histogram [21]. After that, Xiao et al. constructed another two-dimension histogram, called GLGM histogram using gray level of original image and its gradient magnitude. In [22], a 2-D direction histogram was constructed by using the gray level of original image and the orientation of gradient. Zheng et al. constructed a two-dimension histogram using gray level of original image and its local variance [23].

Motivate by the idea of the mentioned works, a new two-dimension histogram construction method is proposed in this paper. The proposed method constructs a new two dimension histogram using gray level of a pixel and it’s local relative entropy of it’s neighbors. Then, the ideal thresholding vector is selected by minimizing a relative entropy based criterion function.

## Entropy and relative entropy

Originally, entropy is a thermodynamic concept, which is used to measure the disorder presented in a system. Entropy became a measure of information amount due to Shannon’s work in [24]. Now, entropy is used to measure the uncertainty of a random variable. Suppose *P* = {*p*_1_, *p*_2_, ⋯, *p*_*n*_} and *Q* = {*q*_1_, *q*_2_, ⋯, *q*_*n*_} are two different probability distributions. The Shannon entropy of probability distribution *P* is given as

$$E\left(P\right)=-\sum_{i=1}^{n}p_{i}\text{log}\;p_{i}.$$ (1)

The relative entropy, also called Kullback—Leibler divergence, between *P* and *Q* is defined as

$$D\left(P,Q\right)=-\sum_{i=1}^{n}p_{i}\text{log}(\frac{p_{i}}{q_{i}}).$$ (2)

The relative entropy measures the difference between two distributions *P* and *Q*.

## Gray level-local relative entropy (GLLRE) two dimensional histogram

Let *I*(*x*, *y*)(*x* = 1, 2, ⋯, *M*; *y* = 1, 2, ⋯, *N*) be the brightness of a pixel located at (*x*, *y*) in the image *I*. *I*(*x*, *y*) ∈ {0, 1, ⋯, *L* − 1}. The local relative entropy (LRE) of a pixel (*x*, *y*) in a *n* × *n* neighborhood is calculated as

$$J\left(x,y\right)=\sum_{i=-(n-1)/2}^{(n-1)/2}\sum_{j=-(n-1)/2}^{(n-1)/2}I\left(x+i,y+j\right)\times |\text{log}\frac{I(x+i,y+j)}{\bar{I}\left(x,y\right)}|,$$ (3)

where $\bar{I}\left(x,y\right)$ is the mean gray level value of the pixels in the neighborhood, which is given as

$$\bar{I}\left(x,y\right)=\frac{1}{n^{2}}\sum_{i=-(n-1)/2}^{(n-1)/2}\sum_{j=-(n-1)/2}^{(n-1)/2}I\left(x+i,y+j\right).$$ (4)

Then LRE of each pixel is normalized to 0 between *L* − 1 as

$$J\left(x,y\right)=[\left(J\left(x,y\right)-J_{\text{min}}\right)/\left(J_{\text{max}}-J_{min}\right))]\times L-1,$$ (5)

where *J*_min_ and *J*_max_ are the minimum and maximum of *J*(*x*, *y*). respectively. From Eq (3), it can be seen that LRE measures the difference of brightness of a pixel between the mean brightness of its neighbors. If the brightness of a pixel is similar to it’s neighbors, the LRE is small. On the contrary, the LRE is large. Usually, if a pixel and it’s neighbor pixels belong to the same class, i.e., background or object, then the LRE is small. If a pixel is noise or is edge pixel, then the LRE is large.

To construct two dimensional histogram, one first calculates the number of pixel pairs such that *I*(*x*, *y*) = *i* and *J*(*x*, *y*) = *j*, which is denoted as *n*_*ij*_. GLLRE histogram is the occurrence frequency, which is calculated as

$$p_{ij}=\frac{n_{ij}}{M\times N}.$$ (6)

**Fig 1:**
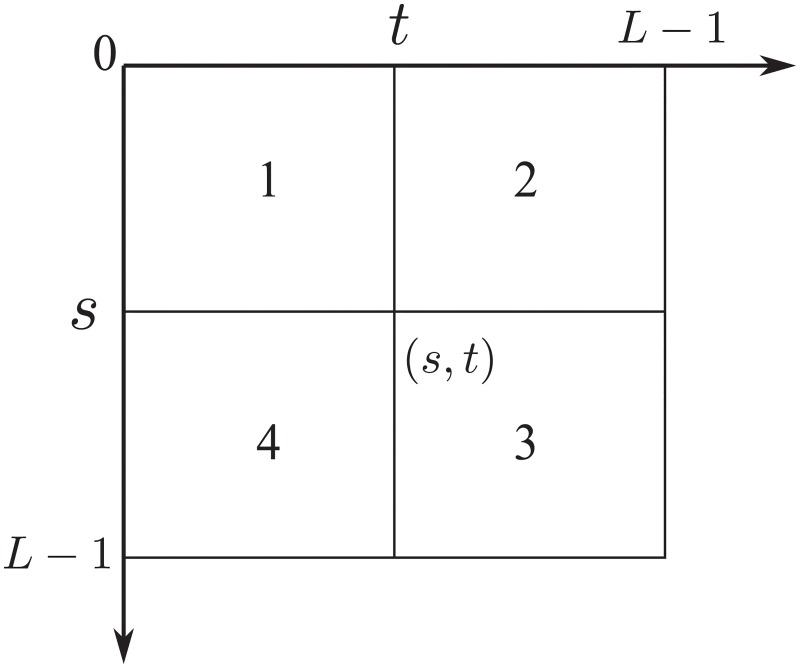
GLLRE two dimensional histogram.

The GLLRE histogram is a two dimensional matrix with size *L* × *L*, which is represented as *P* = {*p*_*ij*_; *i*, *j* = 0, 1, ⋯, *L* − 1}. The GLLRE histogram is shown in Fig 1.

## GLLRRE based thresholding segmentation method

In Fig 1, threshold vector (*s*, *t*) splits the GLLRE into four parts, where *s* is the threshold of original image and *t* the local relative entropy image. As mentioned before, the pixels inside the objects and background have small relative entropy, while the pixels located at edge or noises have large relative entropy. Obviously, parts 1 and 4 represent the objects or background, and parts 2 and 3 be the edges or noises. Let *C*_0_ and *C*_1_ denotes object and background, their probability distribution are

$$C_{0}:\{\frac{p_{ij}}{P_{0}\left(s,t\right)},i=0,1,2,\cdots ,s-1,j=0,1,\cdots ,t-1\},$$ (7)

and

$$C_{1}:\{\frac{p_{ij}}{P_{1}\left(s,t\right)},i=s+1,1,2,\cdots ,L-1,j=0,1,\cdots ,t-1\},$$ (8)

where

$$P_{0}\left(s,t\right)=\sum_{i=0}^{s-1}\sum_{j=0}^{t-1}p_{ij},$$ (9)

and

$$P_{1}\left(s,t\right)=\sum_{i=s}^{L-1}\sum_{j=0}^{t-1}p_{ij}.$$ (10)

To select an ideal threshold vector, an optimization criteria should be determined. In this paper, the minimum relative entropy criteria in [25] is adopted. First, the mean vector of the two classes are calculated as

$$\mu_{0}=\left(\mu_{0i},\mu_{0j}\right)=(\frac{\sum_{i=0}^{s-1}\sum_{j=0}^{t-1}ip_{ij}}{P_{0}\left(s,t\right)},\frac{\sum_{i=0}^{s-1}\sum_{j=0}^{t-1}jp_{ij}}{P_{0}\left(s,t\right)})$$ (11)

and

$$\mu_{1}=\left(\mu_{1i},\mu_{1j}\right)=(\frac{\sum_{i=s}^{L-1}\sum_{j=0}^{t-1}ip_{ij}}{P_{1}\left(s,t\right)},\frac{\sum_{i=s}^{L-1}\sum_{j=0}^{t-1}jp_{ij}}{P_{1}\left(s,t\right)}),$$ (12)

respectively. The two dimensional relative entropy between original image *I* and its segmented version at (*s*, *t*) is calculated as [25],

$$\begin{array}{cc} D(P,Q|,s,t) & =\sum_{i=0}^{s-1}\sum_{j=0}^{t-1}(ip_{ij}\text{log}\frac{i}{\mu_{0i}}+jp_{ij}\text{log}\frac{j}{\mu_{0j}})\\ & +\sum_{i=s}^{L-1}\sum_{j=0}^{t-1}(ip_{ij}\text{log}\frac{i}{\mu_{1i}}+jp_{ij}\text{log}\frac{j}{\mu_{1j}}). \end{array}$$ (13)

The ideal threshold vector (s*, t*) is obtained by minimizing *D*(*P*, *Q*|, *s*, *t*), i.e.,

$$\left(s^{*},t^{*}\right)=\text{arg}\;\text{min}\;D\left(P,Q|,s,t\right).$$ (14)

## Experimental results and discussion

**Fig 2:**
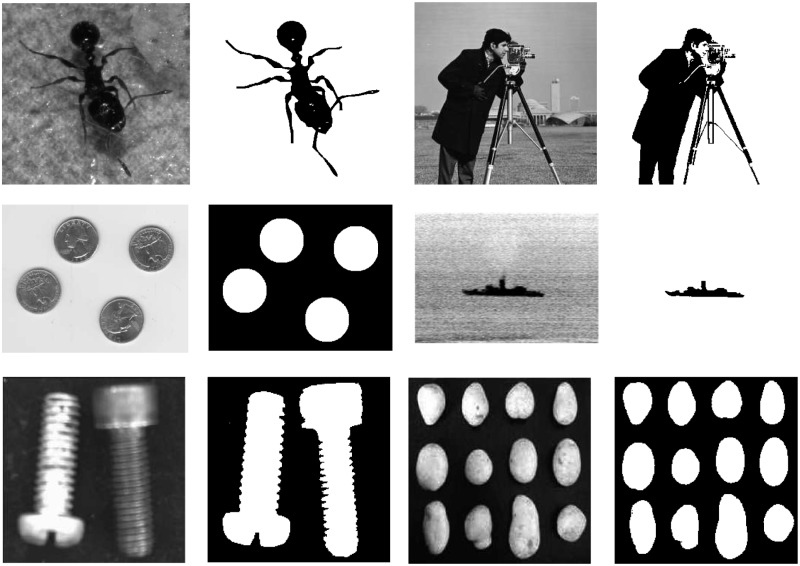
The testing images and their ground-truth images.

In order to illustrate its performance of our proposed method, it is used to segment several images and compared to Otsu thresholding method [14], Otsu method based on GLLRE histogram (Otsu-GLLRE), Kapur method [15] and Kapur method based on GLLRE histogram (Kapur-GLLRE). These methods are implemented on an Intel-i7 3.6GHZ CPU and 8GB memory using Matlab. The images used in the segmentation experiments are *Ant (331×240)*, *Cameraman (256×256)*, *Eight (242×308)*, *Ship (215×302)*, *Skrew(218×219)*, *Stone (244×244)*. All the testing images and their corresponding ground-truth images are shown in Fig 2.

To objectively evaluate the performance of the referenced methods, the misclassification error (ME) is adopted as the evaluation criteria. For bi-level thresholding problem, ME [23] is defined as

$$ME=1-\frac{|B_{o}\cap B_{T}\left|+\right|F_{o}\cap F_{T}|}{|B_{o}\left|+\right|F_{o}|},$$ (15)

where |.| represents the element number of a set, *B*_*o*_ is the set containing background pixels of ground-truth image and *F*_*o*_ containing foreground pixels, *B*_*T*_ is the set containing background pixels in the thresholded image and *F*_*T*_ containing the foreground pixels. ME range from 0 to 1. If ME equals to 0, it implies a perfect segmentation, while equals to 1 for a completely wrong segmentation. The smaller the ME value is, the better our experimental result is.

**Fig 3:**
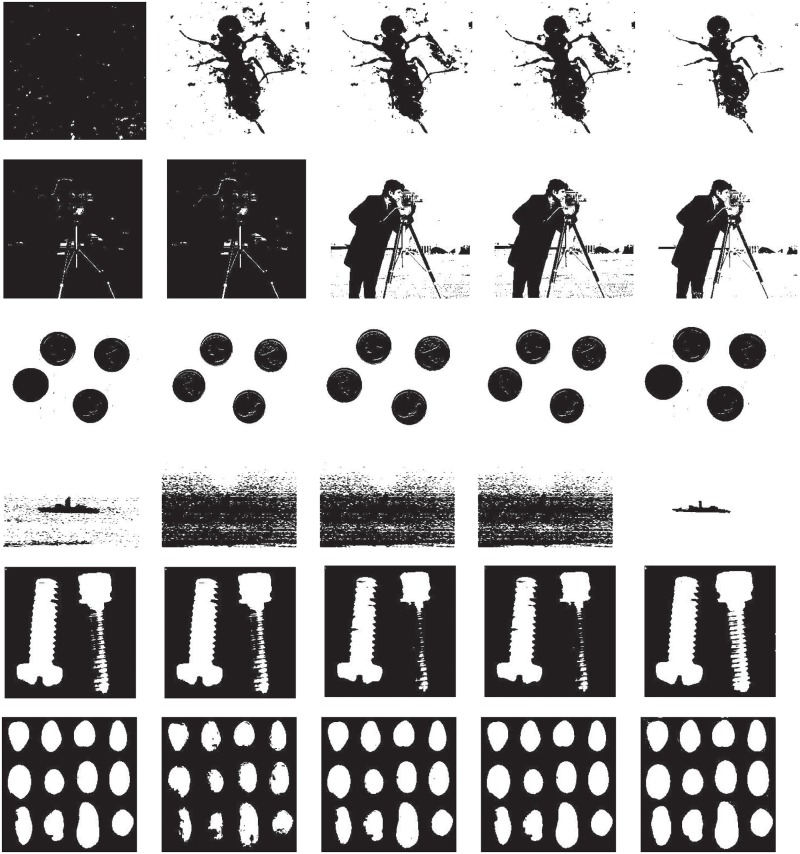
Thresholding results of test image using different methods. From left to right, the results are obtained by Kapur, Kapur-GLLRE, Otsu and Otsu-GLLRE and the proposed method.

The segmentation results are shown in Fig 3. For the *Ant* image, our method achieves the best segmentation result, and *Kapur* method can not separate the object from background, and other three methods exhibit some over-segmentation phnomenon. For *Cameraman* image, whatever Kapur and Kapur-GLLRE method give false segmentation result, while our proposed method produces more accurate segmentation result compared to Otsu and Otsu-GLLRE methods. For *Eight* image, five methods obtain the similar segmentation result. For *Ship* image, there are much pixels in the background are classified as object and results in mistake segmentation for Otsu, Otsu-GLLRE, Kapur and Kapur-GLLRE method. Our proposed method can extract the ship from background. For *Skrew* image, apart from our proposed method, the other four methods can not fully extract the second skrew and under-segmentation phenomenon exists. For *Stone* image, one can see that our method obtain the best segmentation result compared with other referenced methods.

The thresholds or threshold vectors and ME obtained by the referenced methods are listed in Table 1. It can be seen that ME obtained by our proposed method is the smallest, which indicates that our method obtains the best segmentation results.

**Table 1:** The threshold or threshold vector, ME of referenced methods.

Image		Our method	Otsu-GLLRE	Otsu	Kapur-GLLRE	Kapur
ant	threshold	67 242	84 238	84	89 242	183
	ME	**0.0455**	0.0829	0.0829	0.1047	0.8852
cameraman	threshold	69 249	92 249	89	191 249	193
	ME	**0.0224**	0.0301	0.0260	0.7347	0.7367
eight	threshold	212 244	184 244	167	165 244	211
	ME	**0.9588**	0.9763	0.9763	0.9762	0.9600
ship	threshold	24 253	171 253	173	175 253	127
	ME	**0.0024**	0.4812	0.5056	0.5284	0.0566
skrew	threshold	89 253	125 253	126	105 253	105
	ME	**0.0931**	0.1807	0.1824	0.1387	0.1387
stone	threshold	33 252	96 240	106	158 252	87
	ME	**0.0104**	0.0319	0.0390	0.1042	0.0263

## Conclusion

A new method is proposed for image segmentation in this paper. The proposed method is based on GLLRE histogram. GLLRE histogram is constructed by utilizing the brightness and local relative entropy of a pixel and it’s neighbors. The local relative entropy can efficiently measures the brightness difference between a pixel and it’s neighbors. The proposed method integrates the contextual information between pixels into the thresholding process and obtains more accurate segmentation results than other thresholding methods.
